# Intrinsic and extrinsic negative regulators of nuclear protein transport processes

**DOI:** 10.1111/j.1365-2443.2012.01609.x

**Published:** 2012-06-07

**Authors:** Toshihiro Sekimoto, Yoshihiro Yoneda

**Affiliations:** 1 Department of Biochemistry, Graduate School of Medicine, Osaka University1-3 Yamada-oka, Suita, Osaka, 565-0871, Japan; 2 Department of Frontier Biosciences, Graduate School of Frontier Biosciences, Osaka University1-3 Yamada-oka, Suita, Osaka, 565-0871, Japan; 3 JST, CREST, Osaka University1-3 Yamada-oka, Suita, Osaka, 565-0871, Japan

## Abstract

The nuclear–cytoplasmic protein transport is a critical process in cellular events. The identification of transport signals (nuclear localization signal and nuclear export signal) and their receptors has facilitated our understanding of this expanding field. Nuclear transport must be appropriately regulated to deliver proteins through the nuclear pore when their functions are required in the nucleus, and to export them into the cytoplasm when they are not needed in the nucleus. Altered nuclear transport processes have been observed in stressed cells, which would change gene expressions. Some viruses interfere with nuclear transport in host cells to evade immune defense. Moreover, certain transport factors negatively regulate nuclear protein transport in cells. Understanding the regulatory mechanisms of nuclear–cytoplasmic trafficking not only provides important information about cellular processes, but also is of use for developing specific inhibitors for transport pathways.

## Introduction

In eukaryotic cells, the nuclear envelope separates the nucleus and the cytoplasm spatially and functionally. To maintain cell homeostasis, a variety of information is exchanged between the cytoplasm and the nucleus. Genomic information is transcribed to mRNAs in the nucleus and translated to proteins by ribosomes in the cytoplasm. Synthesized proteins are delivered to their target destinations where they function. Nuclear proteins, including structural proteins, transcription factors and other functional proteins must be delivered into the nucleus after their synthesis in the cytoplasm. The proteins that pass through nuclear pores contain a nuclear localization signal (NLS) and a nuclear export signal (NES) within their primary structure. Transport factors recognize and transport cargo proteins containing these signals through the nuclear pore. However, it is noteworthy that not all proteins that function in the nucleus translocate into the nucleus immediately after their synthesis. For example, in the resting state, signal transduction molecules predominantly exist in the cytoplasm, and when cells receive particular stimuli, such signaling molecules are activated and transported into the nucleus. After a certain period of time, in most cases, nuclear export factors return such signaling molecules to the cytoplasm through the nuclear pore. Quantitative analysis estimated that a single proliferating mammalian cell interchanges approximately 1 million macromolecules through nuclear pore within 1-s (Ribbeck & Görlich 2001; Cardarelli *et al*. 2012).

Many regulatory mechanisms concerning the translocation of cargo proteins through nuclear pores have been elucidated. Protein association/dissociation, phosphorylation/dephosphorylation or other modifications of cargo proteins can trigger the nuclear–cytoplasmic transport. Additionally, recent studies showed that other regulatory mechanisms control nuclear–cytoplasmic transport through transport factors and components of nuclear pore complexes (NPCs). Cellular stresses such as UV irradiation and heat shock have been shown to alter nuclear–cytoplasmic transport of proteins. In the field of virology, it has been shown that certain viruses inhibit nuclear–cytoplasmic protein transport specifically or globally in infected cells. Furthermore, some transport factors act as intrinsic negative regulators of certain nuclear proteins. In this review, we focus on the mechanisms and roles of negative regulation of nuclear protein transport, particularly the negative regulation exerted by transport factors themselves.

## Fundamental mechanism of nuclear–cytoplasmic protein transport

To enter the nucleus, molecules must traverse the nuclear envelope via NPC. The NPC is composed of approximately 30 different protein components called nucleoporins (Nups) (Rout *et al*. 2000; Cronshaw *et al*. 2002). It has been proposed that the central channel of the NPC has a mesh-like structure that prevents nonkaryophilic molecules from passing through it freely (Frey *et al*. 2006; Lim *et al*. 2006). Indeed, small molecules such as ions, metabolites and proteins smaller than approximately 40 kDa are able to pass though the NPC diffusely. However, larger molecules require their specific carrier proteins to be transported from the cytoplasm to the nucleus or from the nucleus to the cytoplasm. NLS and NES have been identified in the primary sequences of proteins that are transported through the NPC (Jans *et al*. 2000; Kutay & Güttinger 2005). These signals are recognized by transport factors, called importins and exportins, to be transported into or out of the nucleus (Major *et al*. 2011).

**Figure 1:**
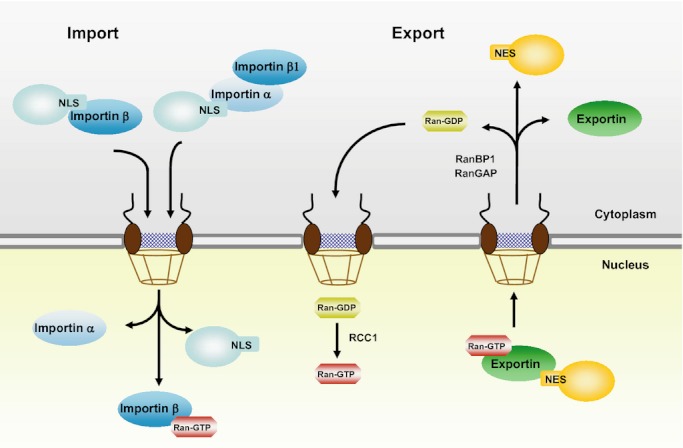
Active nuclear–cytoplasmic transport of proteins. The cargo proteins containing a nuclear localization signal (NLS) are recognized by importin βs alone or importin α/β1 heterodimer, whereas cargo proteins that possess a nuclear export signal (NES) bind to exportins in the presence of Ran-GTP. The transport complexes pass through nuclear pores by sequential and transient interaction of importin β with Nups within the NPC. Within the nucleus, Ran-GTP binding to importin β dissociates the import complex. Ran-GTP to Ran-GDP exchange facilitated by RanBP1 and RanGAP1 dissociates the nuclear export complex in the cytoplasm. Ran-GDP is converted to the GTP-bound form by RCC1 in the nucleus.

A well-known NLS consists of one (monopartite) or two (bipartite) basic amino acid clusters (Table 1). Monopartite or bipartite basic-type NLSs have been identified in many nuclear proteins. Basic-type NLSs are generally recognized by importin α. Importin α transports a cargo protein containing the basic-type NLS in conjunction with importin β1, although importin α has the ability to enter the nucleus with or without its cargo protein in the absence of importin β1 (Miyamoto *et al*. 2002; Kotera *et al*. 2005). However, importin β family proteins can recognize some NLSs directly and transport them into the nucleus without importin α. A cargo–importin complex translocates into the nucleus through transient interaction with phenylalanine–glycine (FG) repeat containing Nups in the NPC (Pyhtila & Rexach 2003). After translocation into the nucleus, the binding of the GTP-bound form of Ran (Ran-GTP) to importin β induces a conformation change that dissociates the importin–cargo complex. The free cargo protein functions in the nucleus, and importins are returned into the cytoplasm by a specific recycling pathway for the next round of transport (Fig. 1).

**Table 1:** Well-known transport signals (NLS and NES) and their specific inhibitors

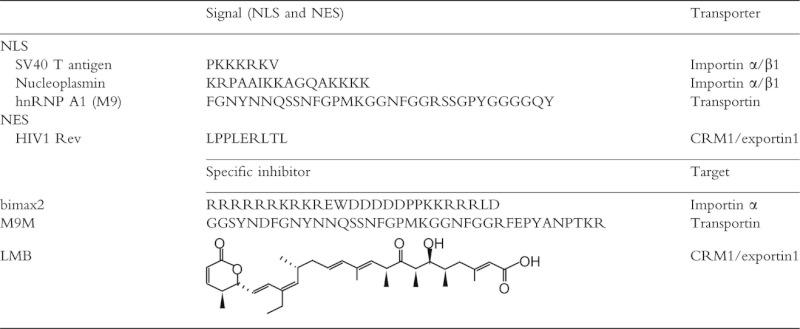

An NES-containing protein is exported from the nucleus into the cytoplasm. For nuclear protein export, the typical NES is characterized as a leucine-rich sequence that is recognized by CRM1/exportin 1 (Table 1). CRM1, a member of the importin β family, binds to Ran-GTP and forms a trimeric complex with the NES-containing cargo protein. This complex translocates through the NPC into the cytoplasm. In the cytoplasm, the conversion of Ran-GTP to Ran-GDP induces the dissociation of the export complex (Fig. 1). In addition to nuclear import, some members of the importin β family act as the NES receptor and transport the NES-containing proteins into the cytoplasm. The importin β family has at least 20 members and transports various proteins in and out of the nucleus. Interestingly, the importin β family is known to recognize protein regions with very diverse sequences, making it difficult to identify its consensus NLS or NES sequence. Low homology within the importin β family may be required to accommodate such divergent NLSs or NESs.

The directionality of protein transport is determined by the gradient of Ran-GTP, which regulates importin β family–cargo interaction. A higher concentration of Ran-GTP in the nucleus than in the cytoplasm has been reported. Ran-GTP induces the dissociation of the import complex and facilitates exportin–cargo binding in the nucleus. The cycling of Ran between GTP- and GDP-bound states is regulated by Ran GTPase-activating protein 1 (RanGAP1), Ran-binding protein 1 (RanBP1) and guanine nucleotide exchange factor RCC1 (Melchior & Gerace 1998; Fig. 1).

## Cellular stresses affect the nuclear transport machinery

Cells are exposed to a variety of environmental stresses such as heat shock, UV irradiation and oxidative stress, but can respond to such stresses through various defense strategies. It has been reported that the classical nuclear import pathway is down-regulated by several stresses (Stochaj *et al*. 2000; Kodiha *et al*. 2004; Miyamoto *et al*. 2004). In the absence of stress, importins and Nups predominantly localize in the cytoplasm and at the nuclear envelope, respectively. After stress treatment, the intracellular ATP level decreases in the stressed cell and the Ran-GTP/Ran-GDP ratio is subsequently reduced, resulting in the cytoplasmic localization of Ran and the down-regulation of Ran-GTP-dependent nuclear export (Yasuda *et al*. 2006). Thus, the decrease in intracellular Ran-GTP induces the accumulation of the NLS receptor importin α and its export factor CAS (cellular apoptosis susceptibility gene) in the nucleus (Miyamoto *et al*. 2004; Kodiha *et al*. 2008a). In addition, oxidative stress by diethyl maleate (DEM) induces the formation of a high molecular mass complex containing importin α, Nup88 and Nup153 in the nucleus, resulting in the retention of importin α in the nucleus (Kodiha *et al*. 2008b). Nuclear importin α accumulated in response to the stresses is less mobile, indicating that importin α recycling from the nucleus to the cytoplasm is inhibited in the stressed cells. Consequently, the abnormal distribution of importin α results in a decrease in importin α/β1-dependent nuclear transport of proteins (Furuta *et al*. 2004).

**Figure 2:**
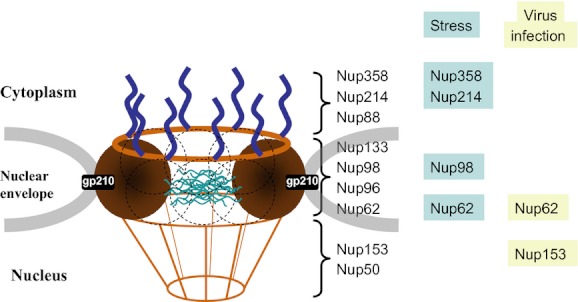
Schematic representation of the mammalian NPC. The relative position of various Nups is listed. The Nups altered by cellular stresses and virus infection are indicated in blue and yellow, respectively.

The NPC exhibits an eightfold symmetry and has a characteristic three-dimensional architecture consisting of approximately 30 different nucleoporins (Nups) (Rout *et al*. 2000; Cronshaw *et al*. 2002; Lim *et al*. 2008; Fig. 2). This architecture is conserved from yeast to higher eukaryotes (Stoffler *et al*. 1999). Transport factors transiently interact with the tandem-repeated phenylalanine–glycine (FG repeats) in Nups to pass through the NPC (Pyhtila & Rexach 2003). Therefore, the appropriate composition of Nups is required for the proper function of the NPC. Cellular stress alters the localization and modification of certain nucleoporins (Fig. 2). Following oxidative stress, the amount of Nup358 is reduced at the nuclear envelope, and Nup98 is redistributed from the nuclear envelope to the nucleoli (Crampton *et al*. 2009). Furthermore, Nup62, Nup98 and Nup214 are phosphorylated, and *O*-linked *N*-acetylglucosamine modification is increased in Nup62 and Nup214 by stress treatment (Crampton *et al*. 2009). These modifications affect CRM1 binding to the NPC and are correlated with a decrease in nuclear protein export. Thus, not only importin α/β1- and CRM1-dependent pathways, but also other transport factor-mediated nuclear transport pathways would be impaired by stress treatment, indicating that cellular stress acts as a negative regulator of nuclear–cytoplasmic transport.

## Viral strategies for their proliferation in host cells

In response to viral infection, signal transduction molecules translocate into the nucleus where they activate the transcription of antiviral genes. On the other hand, to replicate the virus particle in the host cell and to expand its infection to neighboring cells, the virus has various strategies to prevent the antiviral response of the host cell. One of the strategies is to inhibit the nuclear–cytoplasmic protein transport of the host cell. DNA viruses must use nuclear–cytoplasmic protein transport pathways because they replicate in the nucleus of host cells. In contrast, some RNA viruses, which do not use nuclear components of the host cell for their replication, develop strategies to evade host immune defenses by interfering with the nuclear–cytoplasmic protein transport.

**Figure 3:**
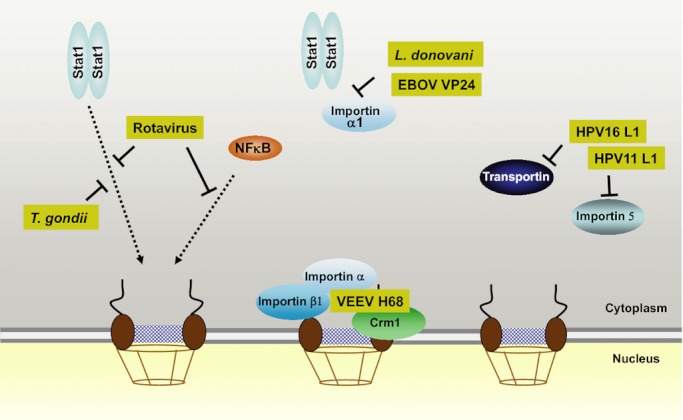
Some viruses and protozoans inhibit the nuclear–cytoplasmic protein trafficking of the host cell. The nuclear import of immune response factors such as Stat1 and NFκB is the target of certain viruses and protozoans. VEEV H68 inhibits both importin α/β1-mediated nuclear import and CRM1-dependent nuclear export pathways. Transportin- and importin 5-mediated nuclear imports are impaired by HPV L1 protein.

It is well known that interferon (IFN) induces antiviral activity. Studies of intracellular signaling by the IFN led to characterization of the Jak–Stat pathway. For example, in response to IFN-γ, interferon receptor–associated Janus kinase 1 (Jak1) and Jak2 are activated and phosphorylate signal transducer and activator of transcription 1 (Stat1; Levy & García-Sastre 2001). NPI-1/importin α2 binds to the tyrosine-phosphorylated Stat1 and transports it into the nucleus in conjunction with importin β1 (Sekimoto *et al*. 1997). After nuclear translocation, Stat1 activates the transcription of numerous genes including those encoding antiviral proteins (Levy & García-Sastre 2001). It has been shown that VP35 protein of Ebola virus (EBOV) has an ability to block the cellular production of IFN-α/β (Basler *et al*. 2000; Reid *et al*. 2005). Furthermore, EBOV also prevents cellular responses to IFN-α/β or INF-γ (Harcourt *et al*. 1999; Kash *et al*. 2006). The minor matrix protein VP24 of EBOV was found to bind to importin α2 and to prevent the Stat1 binding to importin α2, resulting in the cytoplasmic localization of phosphorylated Stat1 (Reid *et al*. 2006, 2007; Mateo *et al*. 2010; Fig. 3). Cytoplasmic Stat1 cannot activate transcription, and the cellular response to virus infection is therefore suppressed. Furthermore, it was found that protozoan parasites have similar negative regulatory activity against the nuclear transport of host cells. *Leishmania donovani* is parasitic in macrophages and evades the host cell's microbicidal properties (Kima 2007). *Leishmania donovani* amastigotes in the host macrophage inhibit the association of Stat1 with importin α2 in response to IFN-γ, causing reduced nuclear localization of activated Stat1 (Matte & Descoteaux 2010). In addition, although the precise mechanisms are unclear, it has been reported that infection by measles virus (MV-N), rotavirus and protozoan parasite *Toxoplasma gondii* also blocks the nuclear translocation of immunoresponsive proteins such as activated Stat1, Stat2 and NFκB (Lüder *et al*. 2001; Holloway *et al*. 2009; Takayama *et al*. 2012).

The L1 major capsid proteins of human papillomavirus (HPV) types 11 and 16 have been reported to inhibit the Kapβ2/transportin- and Kapβ3/importin 5-mediated pathways (Nelson *et al*. 2003l Fig. 3). The L1 protein strongly associates with transportin, and this complex cannot be dissociated by the addition of the M9 sequence, a well-known substrate for transportin (Pollard *et al*. 1996), as well as Ran-GTP, the role of which is to dissociate nuclear import complex. Kapβ3 is known to be a target for both HPV16 E5 oncoprotein and the nonstructural protein 5A (NA5A) of hepatitis C virus, and these viral proteins may also inhibit the Kapβ3-mediated transport pathway (Chung *et al*. 2000; Krawczyk *et al*. 2008).

Venezuelan equine encephalitis virus (VEEV) capsid protein has a unique inhibitory activity against the nuclear transport of the host cell (Atasheva *et al*. 2010). The short stretch containing both NLS and NES sequences inside the VEEV capsid protein, named H68, efficiently inhibits the cellular transcription of the full-length capsid protein (Atasheva *et al*. 2008). H68 shows a strong inhibitory activity toward the importin α/β1-dependent nuclear import pathway *in vivo*. In addition, it also prevents the nuclear export pathway mediated by CRM1 (Atasheva *et al*. 2010). Interestingly, the H68 sequence forms a tetrameric complex with importin α/β1 and CRM1, and this complex formation is essential for the inhibition of nuclear–cytoplasmic transport (Fig. 3). Indeed, the nonpathogenic type of VEEV contains mutations within this sequence, and the pathogenesis of this virus is closely correlated with this sequence. In addition, infection of rhinovirus induces degradation of Nup153 and p62, components of the NPC, resulting in the inhibition of nuclear protein import of the host cells (Gustin & Sarnow 2002; Fig. 2). It has also been reported that the matrix protein of vesicular stomatitis virus (VSV) inhibits bidirectional nuclear transport by interacting with the components of NPC or NPC-associated factors that participate in nuclear–cytoplasmic transport (Petersen *et al*. 2000).

Thus, inhibition of both the nuclear import of immune response molecules and the nuclear export of mRNAs for their target genes also impairs the ability of cells to defend against viral infection. Therefore, some virus proteins function as negative regulators of nuclear–cytoplasmic transport that allow the virus to escape the cellular immune response, raising the idea that these viral inhibitory activities might be a good target for drug discovery.

## Post-translational modifications as negative regulators

**Figure 4:**
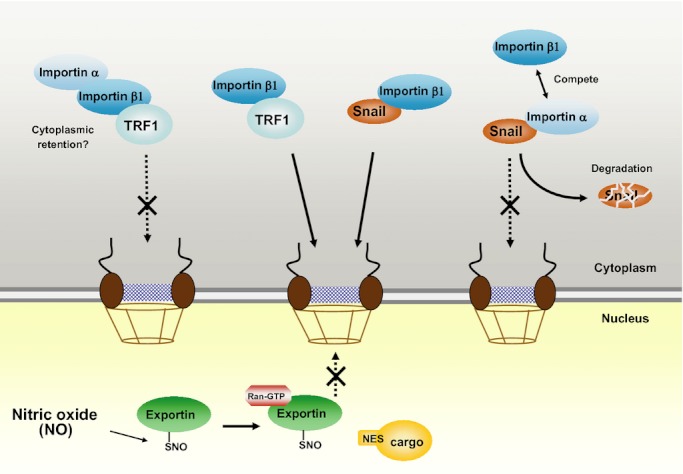
Importin α acts as a negative regulator for the nuclear import of certain proteins. TRF1 and Snail are transported into the nucleus by the importin β1-mediated pathway. In the presence of importin α, TRF1 forms a complex with importin α/β1 heterodimer by binding to importin β1, but this complex remains in the cytoplasm. In contrast, importin α competes with the binding of importin β1 to Snail zinc finger domain, resulting in the inefficient nuclear accumulation of Snail. S-nitrosylation of CRM1 by NO inhibits its binding to NES-containing cargos.

In general, protein modifications are key regulators of protein function, and several modifications have been reported to affect the function of nuclear transport factors. Nitric oxide (NO) has been implicated in regulating the nuclear transport of a number of proteins modified by S-nitrosylation (Buckley *et al*. 2003; Hara *et al*. 2005; Qu *et al*. 2007). NO generated by nitric oxide synthase (NOS) reacts with intracellular glutathione to form nitrosoglutathione, which can interact with a susceptible cysteine residue of proteins, resulting in S-nitrosylation. S-nitrosylation modulates protein functions and is thought to be a major posttranslational regulation (Hess *et al*. 2005). Wang *et al*. (2009) found that CRM1/exportin 1 becomes S-nitrosylated after exposure to NO. Two cysteine residues located in the conserved region of CRM1 are reactive to NO. S-nitrosylation at either cysteine residue is sufficient for the repression of CRM1-dependent nuclear export (Wang *et al*. 2009; Fig. 4). In addition, screening of S-nitrosylated proteins in prostate epithelial cells identified importin β1 as an S-nitrosylated transport factor (Lam *et al*. 2010), although its effects on nuclear transport are still unclear.

In addition to S-nitrosylation, the phosphorylation of transport machinery has been implicated to reduce nuclear–cytoplasmic protein transport (Kehlenbach & Gerace 2000). Importin α/β1- and transportin-mediated nuclear import, but not CRM1-dependent nuclear export, is decreased by treatment with phosphatase inhibitors (okadaic acid and microcystin). The cargo–importin α binding and the interaction of importin β1 with Nup358 and Nup153 were not affected by the phosphatase inhibitors. These findings suggest the possibility that other modifications might negatively control the transport factors or Nups.

## Transport factors as negative regulators

Interestingly, it has been reported that importin α acts as an inhibitor of nuclear protein import in some cases. It is known that importin α acts as an adaptor between an NLS-containing cargo protein and importin β1. Although parathyroid hormone-related protein (PTHrP) and telomere repeat factor 1 (TRF1) are directly recognized and transported into the nucleus by importin β1 alone, PTHrP and TRF1 also form a complex with importin α/β1 heterodimer (Lam *et al*. 1999; Forwood & Jans 2002). Importin α does not directly associate with TRF1, whereas importin α/β1 complex sufficiently binds to TRF1 through importin β1. In contrast to other cargo proteins, this ternary complex is not active and is localized in the cytoplasm (Lam *et al*. 1999; Forwood & Jans 2002). The formation of similar transport-incompetent complexes containing importin α was reported in importin 7/importin β1-mediated nuclear transport of histone H1 (Jäkel *et al*. 1999). It is possible that importin α associates with cytoskeletal components to retain the transport complex in the cytoplasm (Forwood & Jans 2002).

Moreover, importin α directly inhibits the nuclear import of Snail, a zinc finger-containing transcription factor. The zinc finger domain of Snail functions as an NLS, and importin β1 directly recognizes this region and transports it into the nucleus (Yamasaki *et al*. 2005). Importin α also interacts with the Snail zinc finger domain and competes with the binding of importin β1, resulting in the reduced nuclear import of Snail and the induction of its proteasome-dependent degradation (Sekimoto *et al*. 2011; Fig. 4). Importin α acts as a negative regulator for the nuclear import of PTHrP, TRF1 and Snail, although the mechanisms are apparently distinct. Thus, the identification of increasing numbers of targets for the negative regulation mediated by importin α will help to understand more precisely how nuclear transport is inhibited by importin α and its biological significance.

## Small molecules and peptides as negative regulators

Specific chemical inhibitors are powerful tools to study the biological functions of their target molecules. Many chemical compounds have been developed to inhibit specific molecules; in fact, they have contributed to unveiling the details of a variety of biological processes. A well-known inhibitor in the field of protein transport is leptomycin B (LMB), which was originally isolated from *Streptomyces* sp. as an antifungal agent (Hamamoto *et al*. 1983). LMB specifically inhibits a broad range of nuclear protein export pathways mediated by CRM1/exportin 1 (Fornerod *et al*. 1997; Fukuda *et al*. 1997; Kudo *et al*. 1997). It binds covalently to a cysteine residue located in the central conserved region of CRM1 and prevents the binding of CRM1 to proteins containing an NES (Kudo *et al*. 1999). Because of its specific ability to bind to CRM1, LMB is used to determine whether a target molecule is exported from the nucleus by CRM1. The myxobacterial cytotoxin, ratjadone, has been identified as a potent inhibitor of CRM1 (Köster *et al*. 2003). Like LMB, ratjadone covalently binds to the same cysteine residue of CRM1 and inhibits the cargo protein binding to CRM1 (Meissner *et al*. 2004). LMB and ratjadone are structurally related, and their inhibitory activities are similar (Köster *et al*. 2003). Prostaglandin 15-deoxy-D^12,14^-PGJ_2_ was also reported as an inhibitor of CRM1, although it requires a higher concentration (3 orders of magnitude greater than LMB) to block CRM1 (Hilliard *et al*. 2010). Although many chemical inhibitors have so far been developed to block CRM1/exportin 1-dependent nuclear export pathways (Daelemans *et al*. 2002; Van Neck *et al*. 2008; Monovich *et al*. 2009; Mutka *et al*. 2009), small molecule inhibitors for other exportins have not yet been identified.

Several small molecule inhibitors of the nuclear import pathway have been reported. Karyostatin 1A identified by screening for chemical compounds binds specifically to importin β1. It inhibits the interaction of importin β1 with Ran-GTP, resulting in the failure of the classical importin α/β1 pathway *in vivo* and *in vitro* (Hintersteiner *et al*. 2010). A small peptide mimetic inhibitor specific to the importin α/β1 pathway was also developed; however, its potency is low with an IC_50_ value of 106 μm (Ambrus *et al*. 2010). Furthermore, hematoxylin is known to be a pathway-specific inhibitor for the nuclear import of human immunodeficiency virus type 1 (HIV1) Vpr (Suzuki *et al*. 2009). In the presence of hematoxylin, the interaction between importin α and HIV1 Vpr NLS is inhibited in a dose-dependent manner, whereas nuclear import of classical NLS-containing cargos is not affected by hematoxylin (Suzuki *et al*. 2009). Although a micromolar concentration of hematoxylin is required to inhibit the HIV1 replication in macrophages, the discovery of specific inhibitors of viral proteins that affect nuclear transport hints at a new strategy for antiviral drug discovery.

Particular NLS or NES sequence peptides have been used to inhibit specific nuclear transport pathways. Peptide inhibitors are thought to have higher affinities for their targets than small molecule inhibitors, because they can interact with a larger contact area of their target molecules compared with small molecules. NFκB NLS-derived peptide inhibitors (SN50 and SN52) with cell-permeable sequence block the cargo–importin α association and prevent the expression of inflammatory genes by inhibiting NFκB pathways (Lin *et al*. 1995; Xu *et al*. 2008). Similar NLS- or NES-based inhibitors have been reported (Flint *et al*. 2005; Kaiser *et al*. 2009).

These peptides can act as competitors for specific transport pathways, but a high dosage is required for effective inhibition. Therefore, much effort has been made to design high-affinity peptide inhibitors specific to certain nuclear–cytoplasmic transport pathways. Bimax series of peptides are specific inhibitors of the classical importin α/β1 transport pathway (Kosugi *et al*. 2008). Two peptides named Bimax1 and Bimax2 were developed by systematic mutational analysis combined with activity-based profiling. These peptides bind to importin α very tightly with *K*_d_ values in the picomolar range, which is 100 times higher than the affinity of the SV40 NLS (Kosugi *et al*. 2008). Thus, Ran-GTP cannot dissociate the importin α–Bimax complex, resulting in the inhibition of nuclear import pathway mediated by classical importin α/β1.

In addition, a specific inhibitor of the importin β family member was also developed. The hnRNP A1 NLS, called M9, is recognized by transportin/Kapβ2 (Pollard *et al*. 1996). Structure-based design allowed the development of a high-affinity peptide specific to transportin, named M9M (Cansizoglu *et al*. 2007). It strongly binds to Kapβ2/transportin (200 times higher than original M9) and decreases the dissociation efficiency by Ran-GTP (Cansizoglu *et al*. 2007). These peptide inhibitors are expected to be used more broadly for studying the biological function of target transport factors.

## Concluding remarks

Proper nuclear–cytoplasmic protein transport is essential to maintain cell homeostasis. While evidence for the negative regulation of nuclear–cytoplasmic protein trafficking has accumulated, the precise mechanisms and physiological significance remain unclear in most cases. However, it is interesting to know how a virus escapes from cellular immune response by inhibiting nuclear–cytoplasmic transport, because viral inhibitory factors will be good targets for antiviral drug. Furthermore, understanding whether only importin α functions as a negative regulator of nuclear protein transport, and precisely what its physiological significance is, will provide new insights into the nuclear–cytoplasmic transport field. In addition, spatiotemporal regulation of the function of nuclear proteins by specific inhibitors will provide a new strategy for regulating cell function and reveal new drug targets for a variety of diseases.
